# Neutrophil-to-lymphocyte ratio predicts early worsening in stroke due to large vessel disease

**DOI:** 10.1371/journal.pone.0221597

**Published:** 2019-08-26

**Authors:** Ki-Woong Nam, Tae Jung Kim, Ji Sung Lee, Soo-Hyun Park, Hae-Bong Jeong, Byung-Woo Yoon, Sang-Bae Ko

**Affiliations:** 1 Department of Neurology, Seoul National University College of Medicine, Seoul, Korea; 2 Department of Neurology, Seoul National University Hospital, Seoul, Korea; 3 Clinical Research Center, Asan Medical Center, Seoul, Korea; University of Ioannina School of Medicine, GREECE

## Abstract

**Background:**

Inflammation plays an important role in atherosclerosis and its complications. Since a dysregulated inflammatory response is associated with early neurological deterioration (END), serum neutrophil-to-lymphocyte ratio (NLR) could be a marker of END as well.

**Aim:**

In this study, we evaluated the relationship between the serum NLR and END in patients with ischemic stroke due to large-artery atherosclerosis (LAA).

**Methods:**

We evaluated consecutive patients with ischemic stroke due to LAA between January 2010 and December 2015. END was defined as an increase ≥ 2 on the total NIHSS score or ≥ 1 on the motor NIHSS score within the first 72 hours of admission. The NLR was calculated by dividing the absolute neutrophil count by the absolute lymphocyte count.

**Results:**

Of the 349 included patients, 18.1% (n = 63) had END events. In multivariate analysis, serum NLR was independently associated with END (adjusted odds ratio, 1.08; 95% confidence interval [1.00–1.16], *P* = 0.043). Time to admission, and in-situ thrombosis and artery-to-artery embolization mechanisms were also significantly associated with END events. In an analysis of the relationship between serum NLR and vascular lesion burden, serum NLR was positively correlated with both the degree of stenotic lesions (*P* for trend = 0.006) and the number of vessel stenosis (*P* for trend = 0.038) in a dose-response manner. We also compared serum NLR by the stroke mechanisms: patients with hypoperfusion or in-situ thrombosis had the highest levels of NLR: however, only those with in-situ thrombosis had significantly higher NLR in the END group compared to the non-END group (*P* = 0.005).

**Conclusions:**

Serum NLR levels were associated with END events in patients with ischemic stroke due to LAA. Since NLR was also closely correlated with the underlying vascular lesions, our results indicated clues for mechanisms of END events.

## Introduction

Inflammation plays an important role in atherosclerosis.[1] Various circulating inflammatory markers show close association with the presence and degree of atherosclerosis, involving carotid artery, coronary artery, and even peripheral artery.[1–3] Additionally, histological findings reveal that invasion and activation of inflammatory cells destabilize atherosclerotic plaque, increasing the risk of subsequent vascular complications (e.g., ischemic stroke, myocadiac infarction).[4–6] Serum Neutrophil-to-lymphocyte ratio (NLR), as a convenient marker of systemic inflammation, is also thought as having association with atherosclerosis development and its complications.[5] Higher serum NLR is associated with an increased risk of cardiovascular, cerebrovascular diseases, or even subclinical atherosclerosis.[4, 5, 7–10]

Patients with ischemic stroke due to large-artery atherosclerosis (LAA) are prone to early neurological deterioration (END), and thus have worse clinical outcomes.[11, 12] In the acute phase after ischemic stroke, circulating neutrophils are immediately recruited to the ischemic areas, where they secret destructive materials (e.g., reactive oxygen species, proteases, matrix metalloproteinase-9, and cytokines) into neural tissues.[7, 13–15] At the same time, relative lymphopenia develops, partly in response to stress-induced corticosteroids, which attenuates IL-10-mediated healing process after stroke.[7, 13–16] Thus, serum NLR seems to be related to the END in LAA-related stroke because of the close relationship with both atherosclerosis itself and the inflammatory response that occurs immediately after ischemic stroke.

In this study, we aimed to evaluate the relationship between serum NLR and END events in patients with ischemic stroke due to LAA. We also assessed the association between serum NLR and underlying vascular lesions to get clues as to the mechanism by which NLR causes END.

## Material and methods

### Patients and population

We performed a retrospective analysis of acute ischemic stroke registry data prospectively collected within 7 days after symptom onset between January 2010 and December 2015. Among a total of 1,552 patients, those with ischemic stroke due to LAA (n = 443) were initially screened using the Trial of Org 10172 in Acute Stroke Treatment classification.[17] To better determine the effect of initial inflammation on END, only the patients who were admitted within 72 hours after symptom onset were included (n = 389). Patients with severe inflammatory conditions at baseline such as a history of malignancy, hematologic disease, or use of immunosuppressant (n = 23), severe hepatic or renal diseases (n = 10), recent infection using antibiotics within two weeks prior to admission (n = 4), and major surgery or traumatic events (n = 1), were excluded.[5] Patients without information on blood cell counts were also excluded (n = 2). Finally, a total of 349 patients were included in the final analyses (S1 Fig). All patients were evaluated and treated according to the protocol of our institution, including brain magnetic resonance imaging (MRI), magnetic resonance angiography (MRA), echocardiography, and laboratory examinations.

The current study was approved by the Institutional Review Board at the Seoul National University Hospital (No. 1009-06-2-332). This study was designed as a retrospective study in which medical records were only reviewed. Thus, informed consent was not needed and even unattainable.

### Clinical assessment

Electronic medical records were reviewed for demographic information and cardiovascular risk factors, including age, sex, body mass index, hypertension, diabetes, hyperlipidemia, current smoking, and previous history of stroke.[5] Clinical factors including time to admission, initial the NIH Stroke Scale (NIHSS) score, use of thrombolytic therapy, and infectious complication were also evaluated. The initial NIHSS score was assessed on a daily basis from admission to discharge by well-trained neurologists who were not involved in this study. Infectious complication was defined as any infection events, including pneumonia, urinary tract infection, and other infections for three days after admission.[18–20] Laboratory examinations, including glucose profiles, lipid profiles, high-sensitivity C-reactive protein (hs-CRP) levels, and complete blood cell counts, were obtained within the first 24 hours of admission.

To measure serum NLR levels, venous samples were collected in a calcium ethylene diamine tetra-acetic acid (EDTA)-coated tube, and were immediately centrifuged (2,000 rpm for 20 minutes at 4°C).[5] Next, the blood cell counts were determined using an auto-analyzer (XE-2100, Sysmex, Kobe, Japan).[5] The NLR was calculated by dividing the absolute neutrophil count by the absolute lymphocyte count, as reported previously.[5]

END was defined as an increase ≥ 2 on the total NIHSS score or ≥ 1 on the motor NIHSS score within the first 72 hours of admission.[21, 22] The END events were assessed based on the review of medical records by two investigators (K.-W.N. and T.J.K.) without information on other laboratory or radiological factors.

### Radiological assessment

All participants underwent MRI and MRA using a 3.0-Tesla MR scanner (Achieva, 3.0T; Philips, Eindhoven, The Netherlands) within 24 hours of admission. Since we included patients with LAA, most patients had stenosis greater than 50% in the relevant vessels. However, for those with branch atheromatous disease, stenosis less than 50% was allowed. Intracranial atherosclerosis (ICAS) and extracranial atherosclerosis (ECAS) were defined as having stenosis greater than 50% in vessels on time-of-flight MRA.[23–25] Burdens of vascular lesions were rated using the degree of stenosis and the number of vessel stenosis. The degree of stenosis was classified into four groups: absent to mild, 0% to 50% of stenosis; moderate, 51% to 70% of stenosis; severe, 71% to 99% of stenosis; and occlusion, complete vessel occlusion. The number of vessel stenosis was classified as absent, single, and multiple lesions, adding up the number of stenotic vessels in the intracranial and extracranial area. The presence of ICAS and ECAS, and the degree of stenotic lesions were assessed only in the relevant vessels of the index stroke. Symptomatic hemorrhagic transformation was assessed using Safe Implementation of Thrombolysis in Stroke-Monitoring Study (SITS-MOST) criteria, defined by large or remote parenchymal intracerebral hemorrhage (type 2, defined as > 30% of the infarcted area affected by hemorrhage with mass effect or extension outside the infarct) combined with neurological deterioration (≥ 4 points on the NIHSS) or leading to death within 24–36 h.[26] The assessment of radiological markers was conducted by two neurologists (K.-W.N. and T.J.K.) only with information on the index stroke lesions, and any disagreements were resolved by discussion with a third rater (S.-B.K.).

**Fig 1:**
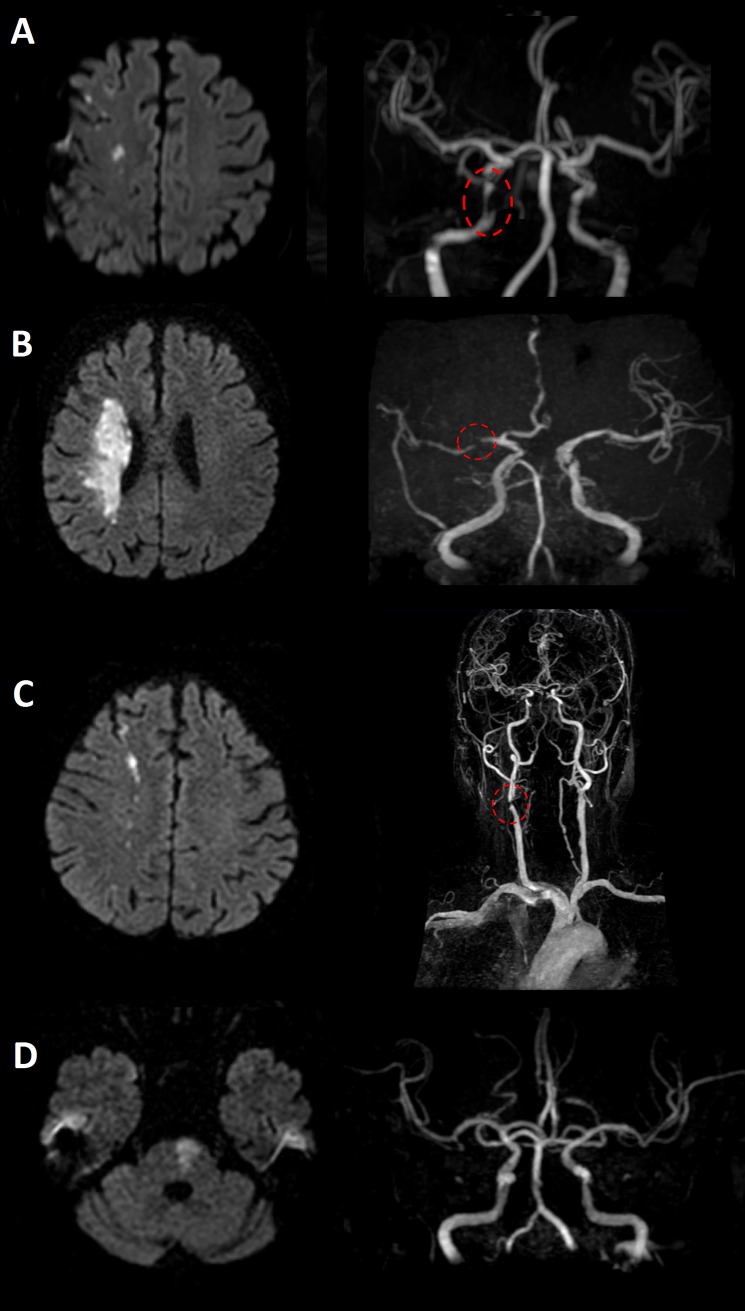
Representative cases of the four types of LAA mechanism. Diffusion-weighted image and time-of-flight MRA images of the four types of LAA mechanism: (A) Artery-to-artery embolization, (B) In-situ thrombosis, (C) Hypoperfusion, and (D) Branch atheromatous disease.

Stroke mechanisms of the index strokes were classified into four groups as described previously[27, 28]: artery-to-artery embolization, in-situ thrombosis, hypoperfusion, and branch atheromatous disease (Fig 1). Artery-to-artery embolization was defined when index diffusion-weighted imaging lesions showed multiple cortico-subcortical infarcts in the same vascular territory with relevant proximal stenotic lesions.[27] In-situ thrombosis was defined when large territorial lesions were found at the location of relevant atherothrombotic lesions on MRA.[27] We classified as hypoperfusion mechanisms in patients with borderzone type infarcts and severe steno-occlusive vascular lesions.[27] Branch atheromatous disease was characterized by an absent-to-mild stenosis on MRA and comma-shaped diffusion-weighted imaging lesions extending to the basal surface of the patent arteries.[27, 29]

### Statistical analysis

All statistical analyses were performed using SPSS version 23 (IBM SPSS, Chicago, IL, USA). In the current study, variables with *P* < 0.05 were considered significant.

#### NLR and END

Univariate analyses for assessing possible predictors of END events were conducted using Student’s *t*-test or the Mann-Whitney *U*-test for continuous variables and the chi-squared test or Fisher’s exact test for categorical variables. Variables with *P* < 0.10 in the univariate analyses and age were introduced into the multivariate logistic regression analysis.[30] To avoid bias from overfitting, several variables which had severe interaction were excluded in the multivariable analysis (e.g., thrombolysis, ICAS, degree of stenosis, and hs-CRP level).

#### NLR and underlying vascular lesions

To obtain clues for the role of NLR in END events, we compared serum NLR levels according to the burden of vascular lesions (e.g., the degree of stenosis and the number of vessel stenosis). The Kruskal-Wallis test and the Jonckheere-Terpstra test were used for these comparisons. Furthermore, we also compared serum NLR values among four different types of LAA stroke mechanisms using the same analytic methods.

## Results

We collected data from 349 patients with ischemic stroke due to LAA (median age, 69 [60–76] years; male, 59.9%; median initial NIHSS score, 3 [2–6]). END occurred in 63 (18.1%) patients. The median time to admission was 12.5 [4–31.5] hours and the median NLR was 2.43 [1.55–3.70] (Table 1).

**Table 1:** Baseline characteristics in END and non-END patients with acute ischemic stroke.

	Total (n = 349)	Non-END (n = 286)	END (n = 63)	*P* value
Age, years [IQR]	69 [60–76]	69 [60–76]	69 [62–78]	0.396
Sex, male (%)	209 (59.9)	172 (60.1)	37 (58.7)	0.836
Time to admission, hours [IQR]	12.5 [4.0–31.5]	15.0 [4.5–31.5]	6.5 [2.5–24.0]	0.003
BMI, kg/m^2^ [SD]	23.7 ± 3.3	23.7 ± 3.1	23.8 ± 4.0	0.815
Hypertension, n (%)	214 (61.3)	170 (59.4)	44 (69.8)	0.125
Diabetes, n (%)	128 (36.7)	108 (37.8)	20 (31.7)	0.370
Hyperlipidemia, n (%)	148 (42.4)	117 (40.9)	31 (49.2)	0.228
Current smoker, n (%)	149 (42.7)	126 (44.1)	23 (36.5)	0.273
Stroke history, n (%)	56 (16.0)	47 (16.4)	9 (14.3)	0.674
Initial NIHSS score [IQR]	3 [2–6]	3 [2–5]	4 [3–7]	0.004
Thrombolysis, n (%)	37 (10.6)	22 (7.7)	15 (23.8)	< 0.001
None	312 (89.4)	264 (92.3)	48 (76.2)	< 0.001
Intravenous thrombolysis	23 (6.6)	16 (5.6)	7 (11.1)	
Intra-arterial thrombectomy	9 (2.6)	4 (1.4)	5 (7.9)	
Both	5 (1.4)	2 (0.7)	3 (4.8)	
Infectious complication, n (%)	26 (7.4)	16 (5.6)	10 (15.9)	0.005
ICAS, n (%)	123 (35.2)	94 (32.9)	29 (46.0)	0.048
ECAS, n (%)	113 (32.6)	96 (33.8)	17 (27.0)	0.296
Stenosis degree, n (%)				0.002
Absent to mild	137 (39.3)	115 (40.2)	22 (34.9)	0.018*
Moderate	60 (17.2)	53 (18.5)	7 (11.1)	
Severe	96 (27.5)	82 (28.7)	14 (22.2)	
Occlusion	56 (16.0)	36 (12.6)	20 (31.7)	
Mechanism, n (%)				0.002
Artery-to-artery embolization	132 (37.8)	118 (41.3)	14 (22.2)	0.005
In-situ thrombosis	52 (14.9)	34 (11.9)	18 (28.6)	0.001
Hypoperfusion	14 (4.0)	11 (3.8)	3 (4.8)	0.724
Branch atheromatous disease	151 (43.3)	123 (43.0)	28 (44.4)	0.835
Number of vessel stenosis, n (%)				0.358
Absent	100 (28.7)	86 (30.1)	14 (22.2)	0.512*
Single	152 (43.6)	120 (42.0)	32 (50.8)	
Multiple	97 (27.8)	80 (28.0)	17 (27.0)	
Symptomatic hemorrhagic transformation, n (%)	3 (0.9)	2 (0.7)	1 (1.6)	0.451
HbA1c, % [IQR]	6.0 [5.7–6.9]	6.1 [5.7–7.0]	5.9 [5.7–6.6]	0.616
Fasting glucose, mg/dL [IQR]	101 [87–124]	100 [87–123]	109 [93–127]	0.099
Total cholesterol, mg/dL [SD]	178 ± 40	177 ± 40	182 ± 37	0.378
Total white blood cells, x 10^3^/μL [IQR]	7.56 [6.40–9.36]	7.53 [6.40–9.26]	7.82 [6.55–9.70]	0.344
Neutrophils, x 10^3^/μL [IQR]	4.67 [3.69–6.42]	4.61 [3.63–6.25]	5.17 [3.86–7.48]	0.056
Lymphocytes, x 10^3^/μL [IQR]	1.99 [1.51–2.60]	2.02 [1.59–2.60]	1.63 [1.20–2.34]	0.003
Platelets, x 10^3^/μL [IQR]	223 [192–262]	223 [193–264]	223 [187–260]	0.711
NLR [IQR]	2.43 [1.55–3.70]	2.31 [1.53–3.48]	3.53 [1.67–5.27]	0.002
hs-CRP, mg/dL [IQR]	0.14 [0.06–0.45]	0.13 [0.05–0.39]	0.27 [0.07–0.94]	0.023

END = early neurological deterioration, BMI = body mass index, NIHSS = National Institutes of Health Stroke Scale, NLR = neutrophil-to-lymphocyte ratio, hs-CRP = high-sensitivity C-reactive protein, ICAS = intracranial atherosclerosis, ECAS = extracranial atherosclerosis

*P values are for linear trends across these variables

In this cohort, high serum NLR was correlated with age, body mass index, hypertension, diabetes, initial NIHSS score, infectious complication, and fasting glucose and total cholesterol levels (S1 Table).

### NLR and END

The baseline characteristics of patients with END were not different to those of patients without END, except for higher initial NIHSS score (4 [3–7] versus 3 [2–5], *P* = 0.004), shorter symptom onset-to-admission time (6.5 [2.5–24.0] hours versus 15.0 [4.5–31.5] hours, *P* = 0.003), and higher rate of thrombolysis therapy (23.8% versus 7.7%, *P* < 0.001) and infectious complication (15.9% versus 5.6%, *P* = 0.005) (Table 1). The END group also had higher percentage of ICAS (46.0% versus 32.9%, *P* = 0.048), and a higher degree of stenosis (*P* for trend = 0.018). Moreover, the END group had higher percentage of in-situ thrombosis (28.6% versus 11.9%, *P* = 0.001), while the non-END group had higher percentage of artery-to-artery embolization (41.3% versus 22.2%, *P* = 0.002). However, the number of vessel stenosis was not different between patients with END and patients without END. In laboratory work up, the END group had a tendency for higher neutrophil counts (5.17 x 10^3^/μL versus 4.61 x 10^3^/μL, *P* = 0.056), but the difference was not statistically significant. However, the END group had lower lymphocyte counts (1.63 x 10^3^/μL versus 2.02 x 10^3^/μL, *P* = 0.003), higher NLR (3.53 [1.67–5.27] versus 2.31 [1.53–3.48], *P* = 0.002), and higher hs-CRP (0.27 [0.07–0.94] versus 0.13 [0.05–0.39], *P* = 0.023).

To identify the factors associated with END, multivariable logistic regression analysis was performed. In the multivariable analysis, NLR remained a positive predictor for END (adjusted OR [aOR], 1.08; 95% confidence interval [CI], [1.00–1.16]; *P* = 0.043) after adjusting for confounders (Table 2). In addition, time to admission (aOR, 0.98; 95% CI [0.96–0.99], *P* = 0.009) and artery-to-artery embolization mechanism (aOR, 0.043; 95% CI [0.21–0.88], *P* = 0.021) were inversely associated with END, while in-situ thrombosis mechanism (aOR, 2.26; 95% CI [1.04–4.91], *P* = 0.041) was positively associated with END, independently of NLR (Table 2).

**Table 2:** Multivariable analyses of the relationship between early neurological deterioration and inflammatory markers with/without stratification by stroke mechanism.

	Crude OR (95% CI)	*P* value	Adjusted OR (95% CI)	*P* value
Age	1.01 [0.98–1.03]	0.579	1.01 [0.98–1.03]	0.587
Time to admission	0.98 [0.96–0.99]	0.009	0.98 [0.96–0.99]	0.009
Initial NIHSS score	1.04 [0.99–1.09]	0.129	0.97 [0.91–1.04]	0.369
Fasting blood sugar (per 10 units)	1.03 [0.97–1.11]	0.334	1.01 [0.93–1.09]	0.891
NLR	1.09 [1.02–1.17]	0.012	1.08 [1.00–1.16]	0.043
Mechanism				
Artery-to-artery embolization	0.52 [0.26–1.04]	0.064	0.43 [0.21–0.88]	0.021
In-situ thrombosis	2.33 [1.15–4.70]	0.019	2.26 [1.04–4.91]	0.041
Hypoperfusion	1.20 [0.31–4.58]	0.792	0.98 [0.24–4.01]	0.980
Branch atheromatous disease	Ref	Ref	Ref	Ref
Infectious complication	3.18 [1.37–7.40]	0.007	2.35 [0.81–6.81]	0.115

NIHSS = National Institutes of Health Stroke Scale, NLR = neutrophil-to-lymphocyte ratio

### NLR and underlying vascular lesions

**Fig 2:**
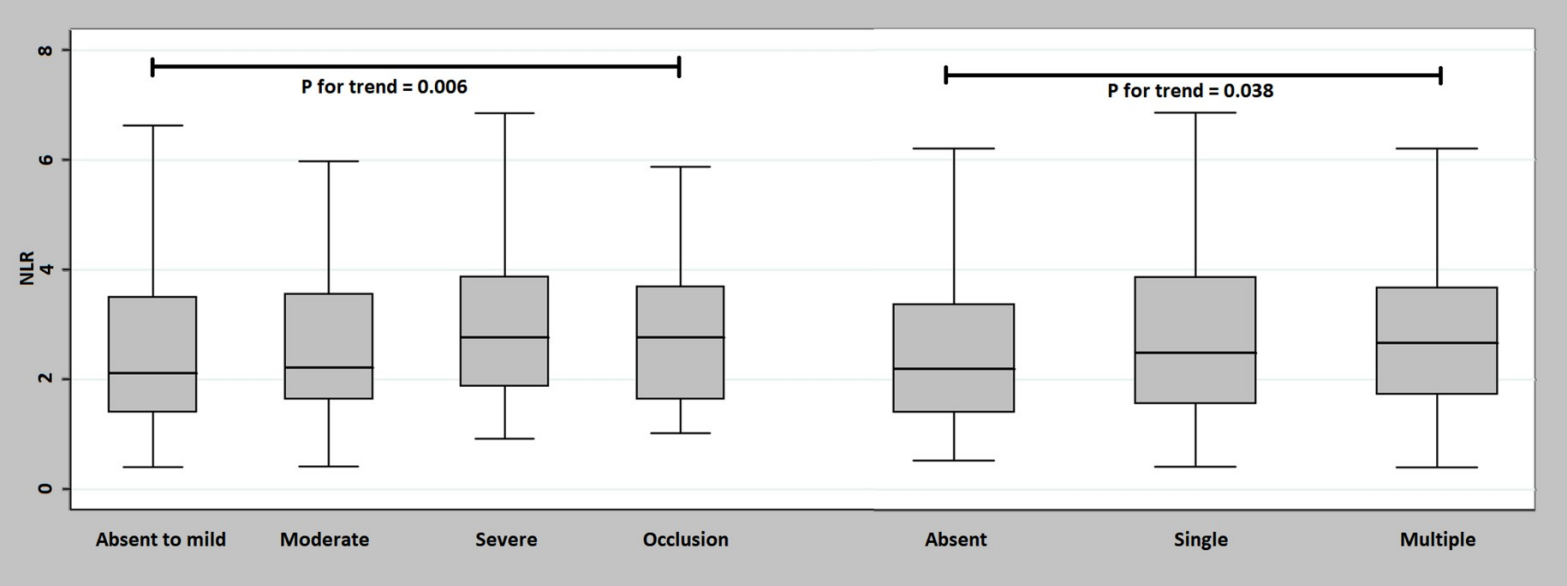
Serum NLR according to the burden of the vascular lesions. Patients with occlusive vascular lesions had the highest NLR levels (*P* = 0.033); serum NLR increased according to the degree of stenosis in a dose-response manner (*P* for trend = 0.006). Furthermore, serum NLR increased according to the number of vessel stenosis in a dose-response manner (*P* for trend = 0.038).

Patients with an increasingly higher degree of stenosis had higher NLR (*P* for trend = 0.006), and those with occlusive vascular lesions had the highest NLR levels (median NLR, 2.76 [1.64–3.70]), suggesting that the burden of the vascular lesions was correlated with inflammatory burden. Furthermore, patients with multiple stenotic lesions also had the highest NLR levels (median NLR, 2.66 [1.74–3.68]) in a dose-response manner (*P* for trend = 0.038, Fig 2).

**Fig 3:**
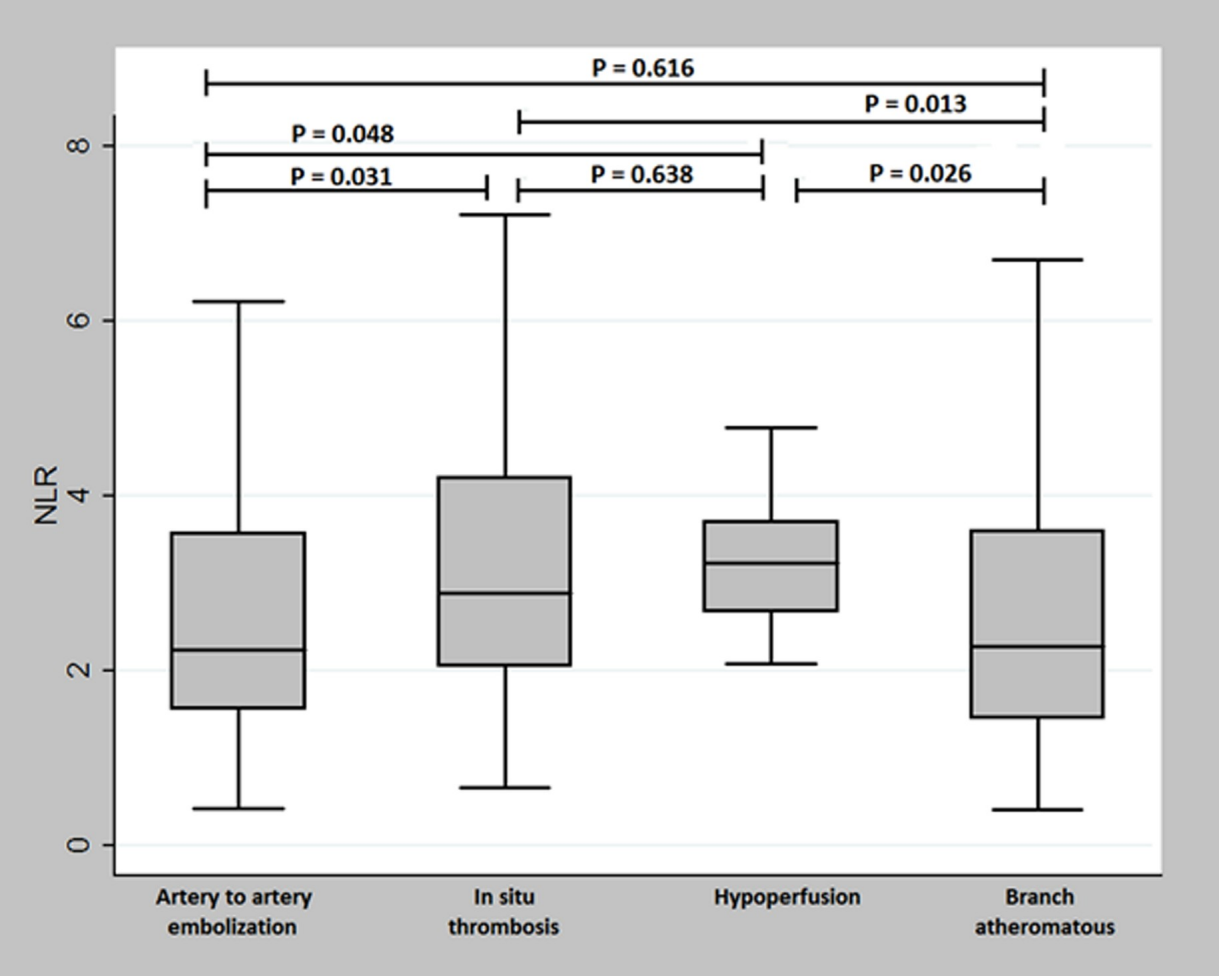
Serum NLR according to stroke mechanism in patients with large-artery atherosclerosis. Patients with in-situ thrombosis or hypoperfusion mechanisms had higher serum NLR levels than those with other stroke mechanisms.

**Fig 4:**
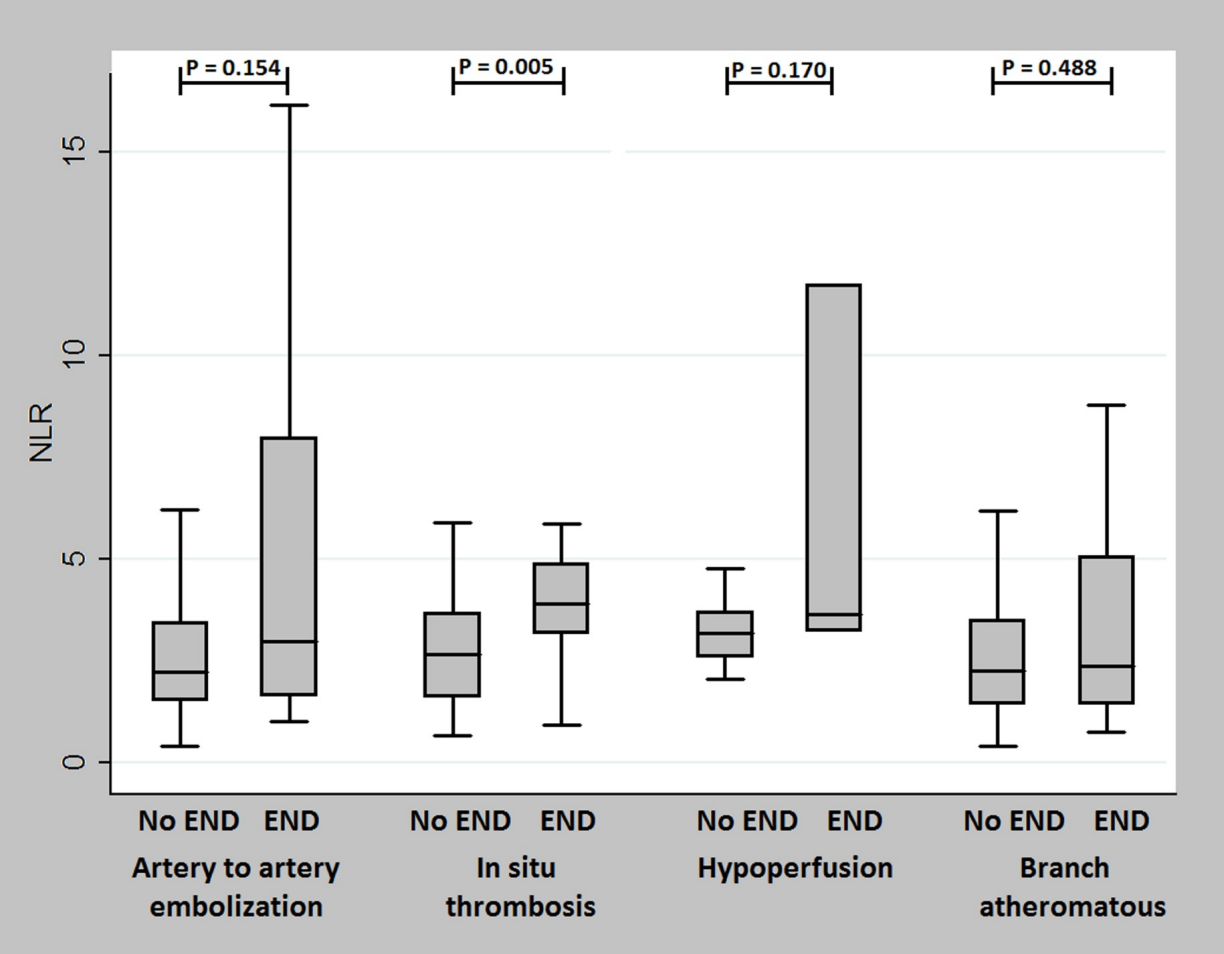
Comparison of serum NLR between patients with END and patients without END by LAA mechanism. Patients with hypoperfusion (median NLR, 3.23 [2.67–3.71]) and in-situ thrombosis (median NLR, 2.88 [2.04–4.21]) mechanisms had higher NLR levels than those with artery-to-artery embolization (median NLR, 2.23 [1.56–3.57]) or branch atheromatous disease mechanisms (median NLR, 2.27 [1.45–3.60]). However, only in-situ thrombosis showed significant differences between the END group and the non-END group (*P* = 0.005).

When we compared NLR values among the four different LAA mechanisms, patients with hypoperfusion (median NLR, 3.23 [2.67–3.71]) or in-situ thrombosis (median NLR, 2.88 [2.04–4.21]) mechanisms showed higher NLR levels than those with artery-to-artery embolization (median NLR, 2.23 [1.56–3.57]) or branch atheromatous disease (median NLR, 2.27 [1.45–3.60]) (Fig 3 and Table 3). However, when we compared between the END group and the non-END group, only the in-situ thrombosis mechanism showed significant differences (*P* = 0.005, Fig 4).

**Table 3:** Characteristics according to stroke mechanism in patients with large-artery atherosclerosis.

	Artery-to-artery embolization	In-situ thrombosis	Hypoperfusion	Branch atheromatous disease
No. of cases	132	52	14	151
NLR	2.23 [1.56–3.57]	2.88 [2.04–4.21]	3.23 [2.67–3.71]	2.27 [1.45–3.60]
hs-CRP	0.14 [0.06–0.48]	0.15 [0.07–0.60]	0.16 [0.10–1.03]	0.13 [0.05–0.41]
Initial NIHSS	3 [1–5]	6 [3–13]	6 [4–9]	4 [2–5]
ICAS	61 (46)	47 (90)	5 (36)	10 (7)
ECAS	89 (68)	6 (12)	11 (79)	7 (5)
Stenosis degree				
Absent to mild	1 (1)	1 (2)	0 (0)	135 (89)
Moderate	41 (31)	6 (12)	3 (21)	10 (7)
Severe	59 (45)	26 (50)	6 (43)	5 (3)
Occlusion	31 (23)	19 (37)	5 (36)	1 (1)
Number of vessel stenosis				
Absent	1 (1)	1 (2)	0 (0)	136 (90)
Single	102 (77)	44 (85)	10 (71)	14 (9)
Multiple	29 (22)	7 (13)	4 (29)	1 (1)

NLR = neutrophil-to-lymphocyte ration, hs-CRP = high-sensitivity C-reactive protein, NIHSS = National Institutes of Health Stroke Scale, ICAS = intracranial atherosclerosis, ECAS = extracranial atherosclerosis

## Discussion

In this study, we found that higher serum NLR was associated with higher risk of END in patients with acute ischemic stroke due to LAA, especially in patients with in-situ thrombosis. Furthermore, NLR levels were closely related to underlying vascular lesion burden.

### NLR and END

The role of NLR as a surrogate marker for inflammation has been previously suggested. Higher NLR is associated with higher incidence of cardiovascular diseases and worse clinical outcome.[9, 31] In previous studies, we showed that NLR can be used as a predictor for initial stroke volume or severity, hypertension, diabetes, atrial fibrillation, and hemorrhagic transformation.[13, 15, 32–34] However, it is still elusive whether NLR can be a risk factor for END in LAA, when END is triggered by inflammation.

The exact mechanism of END among patients with higher NLR is unclear. However, there are several hypotheses. First, higher NLR may simply reflect the severity of the index stroke, and END develops more frequently among patients with severe stroke. Early neutrophilia is common in patients with high severity of the index stroke, and activated neutrophils itself can also negatively affect the infarcted tissues via disruption of the blood-brain barrier, degradation of basal laminar collagen, and formation of hemorrhagic transformation.[7, 13, 15, 16] Our results also showed that serum NLR levels were higher in patients with higher initial NIHSS scores. However, after adjusting for initial stroke severity, NLR level was still strongly associated with the development of END. Therefore, we think that NLR is an independent predictor for END, even though it is a surrogate marker for severe stroke. Second, it could be a simple coincidence that high serum NLR and END have similar risk factors. As shown in S1 Table, patients with higher NLR are vulnerable to ischemic insults with higher burden of cardiovascular risk factors and metabolic diseases, which are also risk factors for END events.[33, 34] However, as mentioned before, NLR level remained significant after adjusting for these risk factors in predicting END. Third, this positive association may depend on the underlying vascular lesions. In general, patients with ischemic stroke due to LAA mechanisms have worse early outcomes,[11, 12, 35] and the burden of stenotic lesions or stability of atherosclerotic plaques seem to have important roles.[11, 27, 35–37] Previous studies have revealed that serum NLR levels are associated with the burden of ICAS and ECAS in a dose-response manner.[4, 5, 10, 38] Furthermore, histological studies have shown that neutrophils are predominantly found in rupture-prone atherosclerotic lesions and are actively involved in plaque destabilization.[4–6, 39] Lymphopenia itself may also be associated with the progression of atherosclerosis due to the lack of a protective role of circulating CD4 T cells. In our patients, serum NLR levels were positively correlated with the degree of stenosis and the numbers of stenotic lesions in a dose-response manner. Although we could not directly evaluate the plaque stability or quantitative plaque volume, we think that serum NLR may reflect underlying plaque burden, which might lead to subsequent END events.

### NLR and underlying vascular lesions

Among the four types of LAA mechanisms, patients with in-situ thrombosis or hypoperfusion had higher NLR levels than patients with artery-to-artery embolization or branch atheromatous disease. However, only patients with the in-situ thrombosis mechanism had higher NLR levels in the END group than in the non-END group. We think that serum NLR, a marker of inflammation, is a sensitive indicator of acute atherothrombosis events with unstable plaques compared to perfusion failure or new embolization. We also found that in-situ thrombosis was an independent risk factor for END events, which is in line with previous studies.[35, 36] In turn, artery-to-artery embolization was negatively correlated with END events. The exact reason for this difference is unclear. However, patients with in-situ thrombosis had more severe initial NIHSS scores, more predominant ICAS lesions, and more severe stenosis, which is more prone to END.[12, 36] Whereas, patients with artery-to-artery embolization had milder index stroke, and mostly ECAS lesions. This discrepancy in the two groups may be a possible explanation for a different risk of END. Otherwise, the effect of inflammation could be more important in patients with ICAS compared to those with ECAS.

### Limitations

This study has several limitations. First, this study was a single-center retrospective observational study. Therefore, the possibility of selection bias should be considered. Second, despite the strong association between NLR and END, our result cannot prove a causal relationship because of the cross-sectional, observational design of the study. Further prospective studies are needed to confirm this relationship. Third, since we included ischemic stroke patients within 72 hours from symptom onset, there is a possibility that END events could be underestimated during the pre-admission period. Lastly, we chose a relatively sensitive definition of END,[40] thus, END events could have been overestimated. However, previous studies using the same definition showed that this END definition can be used as a sensitive definition of END.[21, 22]

## Conclusion

We demonstrated that serum NLR at admission is independently associated with higher plaque burden and END events in patients with ischemic stroke patients due to LAA, especially those with in-situ thrombosis. However, our findings should be validated in larger dataset of patients with more confounders.

## Supporting information

S1 Fig Patient flowchart. (TIF)

S1 Table Univariate linear regression analysis between neutrophil-to-lymphocyte ratio level and baseline characteristics (n = 349). (DOCX)
