# Supplementary figures and images for: Neutrophil-to-lymphocyte ratio predicts early worsening in stroke due to large vessel disease
Source: PLoS One. 2019 Aug 26;14(8):e0221597. doi: 10.1371/journal.pone.0221597 (PMC6709913; doi:10.1371/journal.pone.0221597)

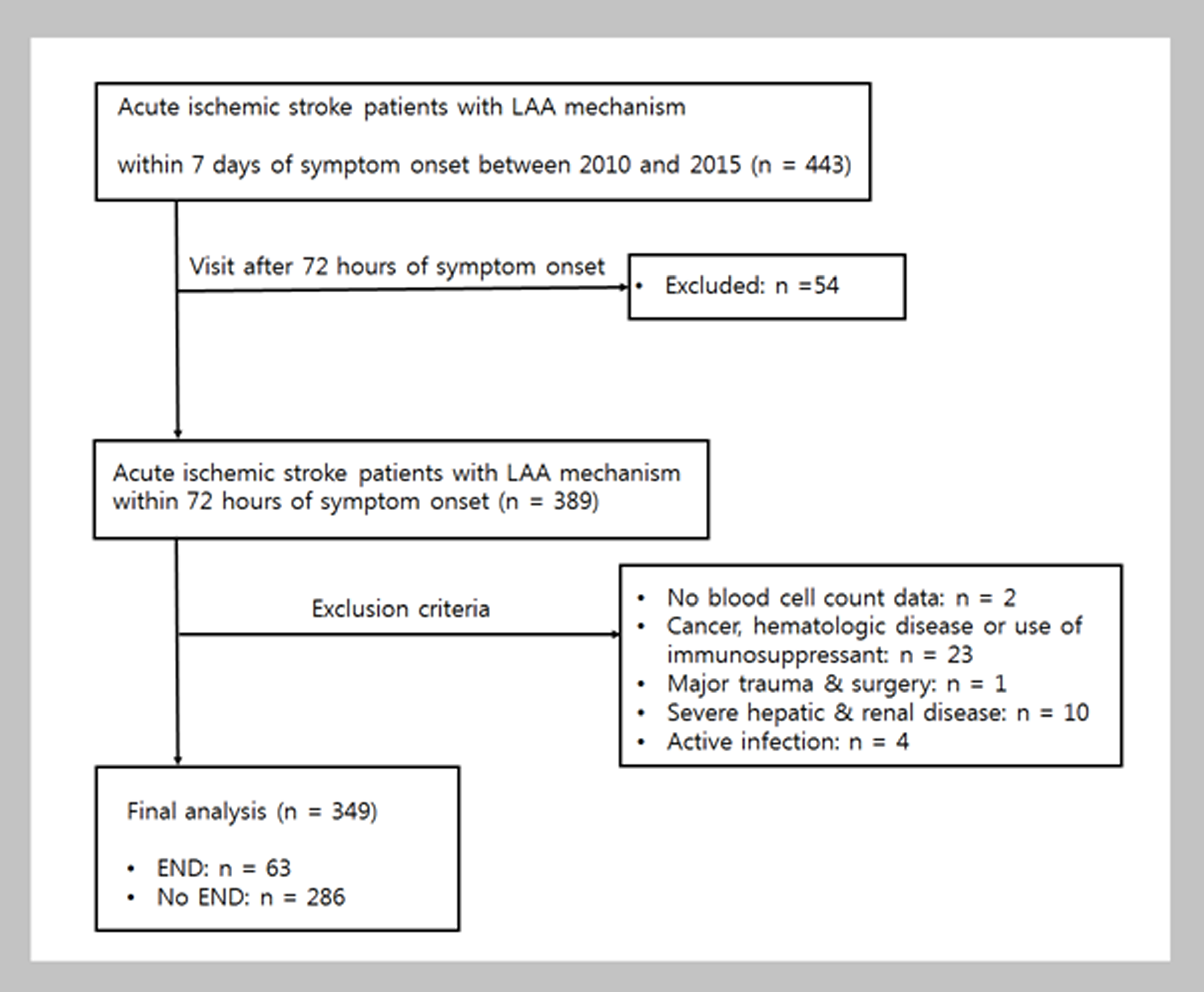

Supplement: S1 Fig — (TIF) [file pone.0221597.s001.tif]
