# Supplementary material for: Neutrophil-to-lymphocyte ratio predicts early worsening in stroke due to large vessel disease
Source: PLoS One. 2019 Aug 26;14(8):e0221597. doi: 10.1371/journal.pone.0221597 (PMC6709913; doi:10.1371/journal.pone.0221597)
Supplement: S1 Table — (DOCX) [file pone.0221597.s002.docx]

**S1 Table. Univariate linear regression analysis between neutrophil-to-lymphocyte ratio level and baseline characteristics (n = 349)**

|  | β (95% CI) | *P* |
| --- | --- | --- |
| Age, years | 0.048 (0.013 to 0.083) | 0.008 |
| Sex, male | -0.262 (-1.095 to 0.572) | 0.537 |
| Visit time, h | -0.009 (-0.029 to 0.010) | 0.350 |
| Body mass index, kg/m^2^ | -0.127 (-0.251 to -0.004) | 0.042 |
| Hypertension | 1.040 (0.208 to 1.872) | 0.014 |
| Diabetes | 1.040 (0.199 to 1.881) | 0.015 |
| Hyperlipidemia | -0.497 (-1.322 to 0.328) | 0.237 |
| Current smoker | -0.403 (-1.229 to 0.422) | 0.337 |
| Stroke history | 0.226 (-0.887 to 1.339) | 0.690 |
| Initial NIHSS score | 0.137 (0.057 to 0.217) | 0.001 |
| Infectious complication | 4.398 (2.912 to 5.884) | < 0.001 |
| Intracranial atherosclerosis | 0.760 (-0.092 to 1.612) | 0.080 |
| Extracranial atherosclerosis | 0.076 (-0.801 to 0.952) | 0.865 |
| Stenosis degree |  |  |
| Absent to mild | Ref | Ref |
| Moderate | 0.281 (-0.901 to 1.463) | 0.650 |
| Severe | 0.499 (-0.528 to 1.505) | 0.345 |
| Occlusion | 0.784 (-0.427 to 1.995) | 0.204 |
| Mechanism |  |  |
| Artery-to-artery embolization | 0.355 (-0.556 to 1.266) | 0.444 |
| In-situ thrombosis | 0.623 (-0.606 to 1.852) | 0.319 |
| Hypoperfusion | 0.679 (-1.456 to 2.814) | 0.532 |
| Branch atheromatous disease | Ref | Ref |
| Number of vessel stenosis |  |  |
| Absent | Ref | Ref |
| Single | 0.597 (-0.383 to 1.577) | 0.232 |
| Multiple | 0.975 (-0.110 to 0.206) | 0.078 |
| Hemorrhagic transformation | 0.326 (-1.179 to 1.830) | 0.671 |
| HbA1c, % | 0.043 (-0.318 to 0.403) | 0.816 |
| Fasting glucose, mg/dL | 0.014 (0.003 to 0.024) | 0.012 |
| Total cholesterol, mg/dL | -0.013 (-0.023 to -0.003) | 0.014 |
